# Multiple Functions of MSCA-1/TNAP in Adult Mesenchymal Progenitor/Stromal Cells

**DOI:** 10.1155/2016/1815982

**Published:** 2015-12-29

**Authors:** David Estève, Jean Galitzky, Anne Bouloumié, Caroline Fonta, René Buchet, David Magne

**Affiliations:** ^1^Inserm UMR 1048, Team 1, Institute of Metabolic and Cardiovascular Diseases, Paul Sabatier University, 1 avenue Jean Poulhès, BP 84225, 31432 Toulouse Cedex 4, France; ^2^CNRS UMR 5549, Brain and Cognition Research Center, Pavillon Baudot, CHU Purpan, BP 25202, 31052 Toulouse Cedex 3, France; ^3^CNRS UMR 5246, Institute of Molecular and Supramolecular Chemistry and Biochemistry (ICBMS), University of Lyon, 43 Boulevard du 11 Novembre 1918, 69622 Villeurbanne, France

## Abstract

Our knowledge about mesenchymal stem cells has considerably grown in the last years. Since the proof of concept of the existence of such cells in the 70s by Friedenstein et al., a growing mass of reports were conducted for a better definition of these cells and for the reevaluation from the term “mesenchymal stem cells” to the term “mesenchymal stromal cells (MSCs).” Being more than a semantic shift, concepts behind this new terminology reveal the complexity and the heterogeneity of the cells grouped in MSC family especially as these cells are present in nearly all adult tissues. Recently, mesenchymal stromal cell antigen-1 (MSCA-1)/tissue nonspecific alkaline phosphatase (TNAP) was described as a new cell surface marker of MSCs from different tissues. The alkaline phosphatase activity of this protein could be involved in wide range of MSC features described below from cell differentiation to immunomodulatory properties, as well as occurrence of pathologies. The present review aims to decipher and summarize the role of TNAP in progenitor cells from different tissues focusing preferentially on brain, bone marrow, and adipose tissue.

## 1. MSCA-1/TNAP Expression in Progenitor Cells

Historically, Friedenstein reported the presence in the adult bone marrow (BM) of cells able to induce bone formation and reconstitute a hematopoietic microenvironment when transplanted subcutaneously [1]. However such a cell population was considered to support indirectly hematopoietic compartment reconstruction by promoting a microenvironment assisting haematopoiesis [2, 3]. By extension from embryonic mesenchymal cells, Caplan coined the term “mesenchymal stem cells” to refer to adult BM precursors [4]. These “mesenchymal stem cells” were later reported to be involved in the formation of various tissues such as bone, cartilage, fat, muscle, ligament, and tendon [5]. In adults, in the stroma of those tissues, only a small fraction can be considered as mesenchymal stem cells, with the capacity of self-renewal while maintaining multipotency. At birth, the frequency of these cells in the BM has been reported as 1 cell/10^4^ BM-mononuclear cells, decreasing to 1 cell/10^5^ BM-mononuclear cells in teenagers to 1 cell/2 × 10^6^ BM-mononuclear cells in 80-year-old individuals [6].

There has been a large semantic confusion in the scientific community, with most researchers using the term “mesenchymal stem cells,” whereas they were working with stromal cells derived from bone marrow or adipose tissue. Consequently, the International Society for Cellular Therapy (ISCT) encouraged the scientific community to use the term “multipotent mesenchymal stromal cell” when stem cell activity was not clearly demonstrated [2, 7]. The following should be considered as MSCs: (1) cells adherent to plastic in culture; (2) cells expressing CD105, CD73, and CD90 [7]. Nevertheless, the ISCT proposed to use the same acronym, MSCs, to abbreviate both “multipotent mesenchymal stromal cell” and “mesenchymal stem cells” [7].

In addition to bone marrow, stromal progenitor cells have been identified in different tissues such as adipose tissue [8], dental pulp [9], and umbilical cord [10], the latter containing the most primitive MSCs. However, differences appear to exist between stromal progenitor cell populations from different tissues in adults based on cell surface marker expression, presenting an additional challenge to devise a universal definition of MSCs [11]. In this context, identifying new cell surface markers in progenitor cells will be helpful to better characterize progenitor cell populations.

### 1.1. The MSCA-1/TNAP Marker

**Figure 1:**
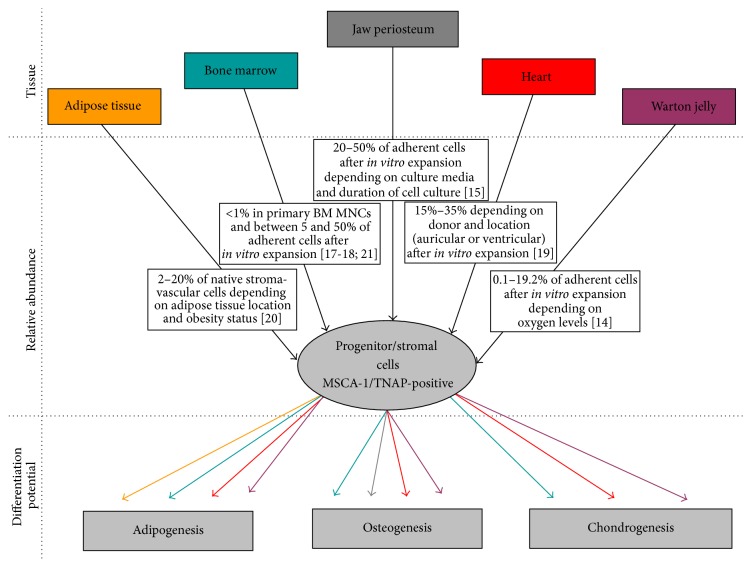
MSCA1/TNAP^+^ cells were described in several tissues (upper panel), with an abundance depending on tissue location and cell protocol (mid panel) and with distinct lineage capacities (lower panel; the line color defines the tissue location). MNC: mononuclear cell.

Two subpopulations have been identified in CD90^+^/CD105^+^ MSCs based on their different expression of a marker recognized by the W8B2 monoclonal antibody, developed by Buhring's group [12]. The protein recognized by this monoclonal antibody was initially defined as the mesenchymal stem cell antigen-1 (MSCA-1). The same group discovered that the protein recognized by the W8B2 antibody is tissue-nonspecific alkaline phosphatase (TNAP) [13]. MSCA-1/TNAP appears to be expressed at the surface of human adult MSCs from several tissues including Wharton jelly, dental pulp, jaw periosteum, and heart and in other species, including bovine and porcine bone marrow MSCs (Figure 1) [14–21]. The fact that TNAP, an ectoenzyme, is expressed in MSCs and ASCs suggests that it plays one or several functions in these undifferentiated cells and/or during their multipotential differentiation. Actually, several functions can be hypothesized in progenitor cells, given that TNAP exerts multiple tasks in different cell types. In humans, TNAP is expressed in multiple tissues, including bone, liver, and kidney. The* ALPL* gene encoding human TNAP is localized on chromosome 1p. It contains 13 exons over 50 kb, the first two exons being part of the 5′-untranslated region (UTR) of the* ALPL* mRNA [22]. Alternative transcription initiation involves either exon 1A or exon 1B in the 5′-UTR [23, 24]. Although species-related differences may exist, transcription of the upstream exon 1A appears preferentially driven by a promoter active in osteoblasts, whereas transcription may be preferentially initiated with exon 1B by a distinct promoter active in liver and kidney [24, 25]. In mouse, the* Akp2* gene encoding TNAP is widely expressed during development. It is expressed first in the neuroepithelium at E8.5 [26] and subsequently in several tissues, such as the biliary canalicula in the liver, growth plate cartilage, bone, and teeth [27].

### 1.2. TNAP Deficiency

In humans and mouse, three important TNAP functions have been identified based on the symptoms of mice and patients with TNAP deficiency. Both* Akp2*-deficient mice and patients with severe hypophosphatasia (HPP), a heritable disease due to defective TNAP function, suffer from bone hypomineralization, abnormalities in brain development including hypodensity of the white matter, dilated ventricles, polycystic encephalopathy, atrophy of the hemispheres, and cortical malformations, and also epileptic seizures [28–32]. The function of TNAP in bone mineralization relies on its ability to hydrolyze inorganic pyrophosphate (PP_i_), a potent inhibitor of calcium phosphate crystal formation [33, 34]. During brain development, TNAP may be required in several processes, including neuron proliferation and differentiation [35] and axonal growth, probably in part through ATP dephosphorylation [36]. Finally, epileptic seizures resulting from TNAP deficiency are likely due to lack of pyridoxal phosphate dephosphorylation in the serum [37, 38], which is an essential step in pyridoxal entry into cells and synthesis of gamma-amino butyric acid (GABA) [39, 40].* Akp2*-null mice die from apnea associated with epileptic seizures before weaning [40, 41], whereas in humans severe forms of HPP are lethal perinatally, due to respiratory complications linked to hypoplastic lungs and rachitic deformities of the chest, apnea [42], and/or to fatal encephalopathy [30, 31]. Early lethality in TNAP-deficient animals has made difficult to determine, whether; TNAP exerts other roles than mineralization, GABA synthesis, and brain development.

### 1.3. TNAP in Liver and Kidney

TNAP is expressed in human hepatocytes, and bile acids increase its activity [43] and secretion in the bile [44]. In addition, TNAP can be delivered from the liver in the serum, where TNAP levels are measured as a marker of cholestasis of major clinical relevance and hepatic dysfunctions [45]. Several TNAP functions in the liver have been proposed, including inhibition of bile secretion [46] and detoxification of lipopolysaccharide (LPS), whose levels are elevated in cholestasis [47]. In human kidney, TNAP is expressed along the proximal tubule in segments S1, S2, and S3 [48], where progenitor cells reside [49]. It remains uncertain today whether TNAP plays a role in PP_i_ metabolism in the kidney and the regulation of renal stone formation (reviewed in [50]) or whether it participates, like in the liver, in LPS detoxification [51].

### 1.4. TNAP and Alzheimer's Disease

In light of several recent reports, TNAP is suspected to play a role in Alzheimer's disease (AD). An increase in TNAP expression and activity has been observed in the hippocampus and serum of AD patients as compared with control subjects [52, 53]. Moreover, TNAP activity is inversely correlated with cognitive functions in AD patients [53]. AD is characterized by the presence of amyloid plaques and neurofibrillary tangles. The plaques are extracellular deposits of amyloid-*β* peptide, whereas the neurofibrillary tangles are intracellular aggregates of the microtubule-associated protein tau, which has become hyperphosphorylated. TNAP seems able to dephosphorylate hyperphosphorylated tau released in the extracellular medium upon neuronal cell death [52] and to amplify neuronal cell death through a robust and sustained intracellular calcium increase due to binding of dephosphorylated tau to muscarinic M1 and M3 receptors [52]. On the other hand, it is also conceivable that TNAP participates in neuronal dysfunction in AD by interfering with the metabolism of ATP and by modulating neuroinflammation. In this context, TNAP inhibition in animal models of AD deserves examination.

### 1.5. TNAP and Vascular Calcification

Vascular calcification is a hallmark of atherosclerosis and type 2 diabetes mellitus but the disease during which arterial calcification has the most dramatic impact is chronic kidney disease (CKD). Indeed, vascular calcification probably represents the leading cause of death in patients with end-stage renal disease [54]. TNAP is activated in arteries of patients with CKD and likely triggers calcification by hydrolysing PP_i_ [55, 56]. In fact, PP_i_ administration prevents vascular calcification in rodent models of CKD [57, 58]. Since PP_i_ treatment may not be a suitable treatment option in humans [59], inhibition of TNAP activity or expression has emerged as a promising strategy to increase life expectancy in patients in end-stage renal disease [60, 61].

Experiments performed in the coming decade will help determine whether TNAP control is an efficient therapeutic strategy in AD and CKD. In parallel, the determination of the mechanisms through which TNAP exerts its effects will be useful to better understand the pathogenesis and development of these diseases. Thorough examination of the growing literature on TNAP functions suggests that TNAP may play at least two important roles in tissue progenitors: cell differentiation and immunomodulation.

## 2. MSCA-1/TNAP and Progenitor Cell Differentiation

TNAP was shown to participate in differentiation of neuron progenitors [35, 36]. TNAP is expressed in neuron membranes in the mouse and human brain [62–64]. A strong and transient expression of TNAP is associated with the earliest stages of morphological differentiation of the neural plate and then serves as a marker of neural progenitors throughout cortical neurogenesis [65]. In adults, a high level of TNAP is maintained in progenitor cells of neurogenic niche (lateral ventricles) where it appears essential for proliferation and differentiation of stem cells [65]. Indeed, TNAP deficiency in mouse delays synapse maturation thus altering axon size [32]. In cultured neurons, TNAP hydrolyses extracellular ATP in neuronal cells and abolishes the ATP inhibition of axonal growth that is mediated by P2X7 receptor activation [36]. Moreover, in search for partners of the cellular prion protein, Ermonval et al. identified TNAP in both serotonergic and noradrenergic neuron cell lines [66]. This latter study showed that onset of functional TNAP accompanies the bioaminergic differentiation of precursor cells and that, during differentiation, induction of TNAP reaches a maximal level upon implementation of a complete serotonergic or noradrenergic phenotype [66].

Besides this large body of evidence indicating a role for TNAP in neuron progenitors, several observations suggest that TNAP may also play a role in differentiation from bone marrow and adipose tissue progenitor cells. Using clones of bone marrow stromal cells, Kim et al. suggested that TNAP activity might favor osteoblast and adipocyte differentiation at the expense of chondrocytes [67]. Slightly different results were obtained by Battula et al. who found that MSCA-1/TNAP-positive cells are able to differentiate into chondrocytes when they also express CD56 (*a.k.a.* neural cell adhesion molecule), whereas adipocytes only emerged from MSCA-1/TNAP^+^ CD56^−^ cells [21]. In this study, osteoblasts could be obtained from both MSCA-1/TNAP^+^ CD56^+^ and MSCA-1/TNAP^+^ CD56^−^ cells [21]. Although no experiment aimed at deciphering the precise role of TNAP in osteoblast differentiation was specifically carried out in human progenitor cells, several reports suggest that TNAP activity induces or accelerates osteoblast differentiation. First,* in vivo* overexpression of TNAP in vascular smooth muscle cells induces expression of osteoblast markers in arteries and triggers vascular calcification [68]. Second, inhibition of TNAP with siRNA, shRNA, or tetramisole (a TNAP inhibitor) in human and mouse osteoblasts reduces expression of osteocalcin, a specific marker of mature osteoblasts [69–71]. ATP could be one of the suspected substrates for TNAP, and recently Orriss et al. described ATP as an inhibitor of bone formation [72, 73]. To date, however, the targets through which TNAP impact osteoblast differentiation remain to be clearly demonstrated.

Besides bone marrow, a native cell population positive for MSCA-1/TNAP was recently described within the stroma-vascular fraction of human adipose tissue [20]. MSCA-1/TNAP was expressed at the membrane of native specific progenitor cell subsets with the highest white and brite (brown in white) adipogenic potential. Furthermore, during adipogenic differentiation, an increase in the expression and the activity of MSCA-1/TNAP was reported while inhibition of its activity by pharmacological or RNA interference approaches led to the inhibition of adipogenesis* in vitro *[20]. Interestingly, among the long list of abnormalities reported in* Akp2* ^−/−^ mice, the lack of adipose tissue [41] reinforces the relevant role of MSCA-1/TNAP in adipogenic differentiation process. Again, the mechanisms through which TNAP stimulates adipocyte differentiation are unknown.

## 3. MSCA-1/TNAP and Immunomodulation in Progenitor Cells

It is today well-known that MSCs exert immunomodulatory functions. Excellent reviews have been published recently to present the known or suspected mechanisms [74–77]. Secretion of several pro- and anti-inflammatory factors has been particularly highlighted [78]. A role for TNAP in MSC-modulated inflammation can be hypothesized since TNAP is modulated by different cytokines in these cells. In human MSCs cultured in osteogenic conditions, TNAP expression and activity are increased by tumor necrosis factor- (TNF-) *α*, interleukin- (IL-) 1*β*, and IL-17 [79–84]. Moreover, the fact that TNF-*α* and IL-1*β* activate TNAP in human MSCs while at the same time they inhibit their osteoblastic differentiation [79] suggests that TNAP may play a specific role in inflammation. Interestingly, cases of chronic recurrent multifocal osteomyelitis have been reported by the groups of Whyte and Girschick in unrelated children with childhood HPP, who suffered chronic, multifocal, periarticular pain and soft tissue swelling. Bone marrow edema was shown by MRI in the metaphyses of long bones, and nonsteroidal anti-inflammatory drugs diminished the clinical features of childhood hypophosphatasia, especially in regard to pain and to the secondary metabolic inflammation resulting from the disease [85–89]. Even though bone inflammation in children with HPP may rely on TNAP function in other cells than bone marrow stromal progenitors, it nevertheless indicates that TNAP loss of function triggers bone inflammation.

In contrast to what was observed under osteogenic conditions, inflammatory cytokines seem to inhibit TNAP activity and consequently white and brite adipocyte differentiation in human adipose progenitor cells (CD34^+^/CD31^−^/CD45^−^) [20]. Indeed TNF-*α* treatment decreases both TNAP expression and activity, while interferon-*γ* and IL-17 (although more slightly) inhibit TNAP activity only. In agreement with an inhibitory effect of inflammation on TNAP in adipose tissue, the number of TNAP-positive cells is lower in visceral compared to matched subcutaneous adipose tissue from obese patients, a depot where the number of immune cells is higher [20]. The reason why one cytokine exerts different effects on TNAP depending on the cell model and lineage (osteoblast versus adipocyte) is not known but deserves investigation.

In progenitor cells, TNAP may modulate the inflammatory response through autocrine and paracrine ways, in particular through adenosine metabolism [90]. ATP can be released or leaked into the extracellular milieu by virtually every cell in the body in response to various inflammatory stimuli. Then, ATP triggers anti-inflammatory signaling cascades through high-affinity P2Y receptors and, at higher concentrations, proinflammatory signaling cascades through low-affinity P2X receptors on the membranes of neighboring cells [91]. Due to their higher affinity, P2Y receptors would likely respond first to any changes in ATP concentration, and immunosuppressive signaling may prevail until amounts of extracellular ATP reach the P2X activation threshold, inducing proinflammatory signaling [91]. Human MSCs are able to release high amounts of ATP [92]. In human MSCs stressed by serum deprivation in culture, ATP acts in an autocrine manner to prevent MSC apoptosis [93]. In addition, ATP appears to increase the production of the proinflammatory cytokines IL-2, interferon-*γ*, and IL-12 in human MSCs, while decreasing the anti-inflammatory cytokine IL-10 [94]. These effects on cytokine production are associated with the reduced ability of MSCs to inhibit T-cell proliferation [94]. ATP inflammatory effects on human MSCs are resolved by at least two ectonucleotidases, CD39 and CD73 [90, 95]. CD39 is an ectonucleoside triphosphate phosphohydrolase that removes two inorganic phosphates (P_i_) from ATP to produce AMP [90]. AMP is then hydrolyzed into adenosine by CD73, which, like TNAP, is a GPI-anchored ecto-5′-nucleotidase expressed in human MSCs [90]. Adenosine receptors are expressed by human MSCs [92], where they likely activate anti-inflammatory signals [96]. In conclusion, the presence of TNAP in MSCs and its capacity to dephosphorylate ATP suggests that, together with CD39 and CD73, TNAP may constitute a molecular triad acting in a coordinated manner to dephosphorylate adenosine nucleotides and control local inflammation.

## 4. Concluding Remarks

Characterization and functions of mesenchymal progenitor cells provide a better understanding on how the cells are able to differentiate and proliferate forming various tissues such as bone, cartilage, and fat. However, two of the difficulties encountered in this type of research are the relatively small amount of mesenchymal progenitor cells in adult tissues and the need to characterize them by specific markers. Here we reported and discussed the characteristics and functions of the marker MSCA-1/TNAP, which was initially identified in progenitor cells with the W8B2 monoclonal antibody [12, 13]. Since TNAP is known for long as a biological marker for mineralizing cells, it accredited the idea that MSCA-1/TNAP mesenchymal cells can differentiate and form bone tissues. This turns out to be true. However, recent findings suggest that TNAP activity within progenitor cells also induces adipocyte and neuron differentiation, as well as immunomodulation. Together, these observations pave the way to important steps that remain to be reached to determine the pathophysiological roles of TNAP and decipher the mechanisms through which TNAP acts.

## Abbreviations

AD: Alzheimer's disease
AMP: Adenosine monophosphate
ASC: Adipose stromal cells
ATP: Adenosine triphosphate
BM: Bone marrow
CD: Cluster of differentiation
CKD: Chronic kidney disease
GABA: Gamma-amino butyric acid
HPP: Hypophosphatasia
GPI: Glycosylphosphatidylinositol
IL: Interleukin
ISCT: International Society for Cellular Therapy
LPS: Lipopolysaccharide
MNC: Mononuclear cell
MSC: Mesenchymal stromal cell
MSCA-1: Mesenchymal stromal cell antigen-1
P_i_: Inorganic phosphate
PP_i_: Inorganic pyrophosphate
TNAP: Tissue-nonspecific alkaline phosphatase
TNF: Tumor necrosis factor
UTR: Untranslated region.
